# Dissecting the Transcriptional Regulatory Properties of Human Chromosome 16 Highly Conserved Non-Coding Regions

**DOI:** 10.1371/journal.pone.0024824

**Published:** 2011-09-13

**Authors:** José Luis Royo, Carmen Hidalgo, Yolanda Roncero, María Angeles Seda, Altuna Akalin, Boris Lenhard, Fernando Casares, José Luis Gómez-Skarmeta

**Affiliations:** 1 Centro Andaluz de Biologia del Desarrollo, CSIC-Universidad Pablo de Olavide-Junta de Andalucía, Sevilla, Spain; 2 Computational Biology Unit, Bergen Center for Computational Science, University of Bergen, Bergen, Norway; 3 Sars Centre for Marine Molecular Biology, University of Bergen, Bergen, Norway; University of Connecticut, United States of America

## Abstract

Non-coding DNA conservation across species has been often used as a predictor for transcriptional enhancer activity. However, only a few systematic analyses of the function of these highly conserved non-coding regions (HCNRs) have been performed. Here we use zebrafish transgenic assays to perform a systematic study of 113 HCNRs from human chromosome 16. By comparing transient and stable transgenesis, we show that the first method is highly inefficient, leading to 40% of false positives and 20% of false negatives. When analyzed in stable transgenic lines, a great majority of HCNRs were active in the central nervous system, although some of them drove expression in other organs such as the eye and the excretory system. Finally, by testing a fraction of the HCNRs lacking enhancer activity for *in vivo* insulator activity, we find that 20% of them may contain enhancer-blocking function. Altogether our data indicate that HCNRs may contain different types of cis-regulatory activity, including enhancer, insulators as well as other not yet discovered functions.

## Introduction

A decade after the release of the first human genome's draft, we do not understand most of the information encoded in these 3 Gigabases of DNA. The degenerated triplets that encode the composition of the proteins impose a constraint in the random potential of DNA sequences which facilitates the prediction of most protein-coding genes. In addition, transcription expression analysis have led the scientific community to extensive knowledge on RNA levels and alternative splicing in different tissues and developmental stages on a variety of animal models. Thus, we can probably assume a successful annotation of most of the protein-coding genes of the higher organisms sequenced so far. However, this knowledge is in striking contrast to our capacity in predicting the existence of cis-regulatory elements, which are embedded in the remaining 98% of the genome. Thus, number, behavior and nature of most regulatory elements governing gene transcription remains poorly determined.

The comparison of all the sequenced vertebrate model organisms revealed the presence of many highly conserved non-coding regions (HCNRs) present in vertebrate genomes [1], [2], [3]. Most of these regions are associated with genes with roles in body patterning and organ morphogenesis [1], [3]. Functional studies using transgenic assays in mouse, *Xenopus* and zebrafish carried out by various groups, indicate that a significant fraction of the HCNRs so far analyzed behave as enhancers in functional assays. These enhancers likely activate the expression of genes essential for embryonic development in specific embryonic domains (see for example [1], [4], [5], [6], [7]. Based on these observations, it has been speculated that the approximately 3000 HCNRs present in all vertebrates likely contain regulatory elements essential for the basic vertebrate body plan [8], [9]. Other initiatives to identify potential cis-regulatory elements are based in chromatin immunoprecipitation experiments coupled to massive sequencing using both transcription factors and epigenetic marks [10], [11], [12], [13], [14], [15], [16], [17], [18], [19]. These studies have enormously expanded the collection of candidate cis-regulatory elements present in the vertebrate and invertebrate genomes. These huge amount of potential cis-regulatory already available, and continuously growing, need to be validated in animal model systems in order to explore their precise *in vivo* temporal and spatial activity. Efforts in this direction are been done using the mouse as a model system [5], [20], [21]. In these studies more than 1000 potential cis-regulatory elements have been assayed by transient transgenic assays in mouse embryos at a single developmental stage. These have lead to the identification of multiple tissue-specific enhancers, many of them evolutionary conserved at the sequence level. These enhancer assays in transient murine transgenics are laborious and expensive and usually limited to a single developmental time point, and therefore not particularly suited for large scale screens. *Xenopus* and zebrafish have been used as alternative models to systematically evaluate *in vivo* de enhancer activity of potential cis-regulatory elements [1], [6], [7], [22], [23], [24]. The development of Tol2 mediated transgenesis in zebrafish [25] the transparency of its embryo and larvae, which is perfect for imaging, and its accessibility to genetic manipulations, makes this animal an ideal model for the *in vivo* analysis of cis-regulatory element activity [26], [27]. Nevertheless, since the generation of stable transgenic lines in zebrafish is time consuming, most middle to large-scale enhancer screenings in zebrafish are based on transient F0 studies [6], [7], [24], [28], [29], [30], [31]. Assays in F0 (i.e. injected) zebrafish have the strong advantage of being a medium throughput approach, but since the integration of the reporter construct occurs only in some cells of the injected embryo, the activity of the potential enhancer is mosaic therefore revealing a fraction of the territory where the regulatory element under evaluation is potentially active. Moreover, enhancer activity can be affected by the regulatory elements in the vicinity of the insertion point (what is commonly known as “position effect”). Recently, the ZED vector was developed [32]. The two major characteristics of this vector is that the reporter cassette is flanked by insulators that reduce the position effect, and that the vector contains a positive control of transgenesis that allows to monitor the efficiently of integration of the transgenic construct both in transient injected and stable transgenic embryos [32].

Here we use the ZED vector to evaluate the activity of more than a hundred HCNR from the human genome, first in transient assays and later in stable transgenic assays in zebrafish. Then, animals showing reporter activity in F0 were grown to adulthood to establish stable transgenic lines in which the enhancer activity was characterized in detail and at different developmental stages. In addition, a collection of injected embryos showing no enhancer activity was grown further to derive stable transgenic lines. Combining the results from these two experiments allowed us to determine the fraction false positive and false negative enhancers. Analysis of the stable transgenic lines allowed us to identify two different categories of enhancers. A first category is that of enhancers that drive consistent, tissue-specific patterns in all the founder lines; a second category is contains elements that stimulate promoter activity, but the precise patterns driven differ among founder lines –likely due to extreme sensitivity to the regulatory information surrounding the insertion point in each founder line. These two types of enhancers have been already described when enhancer activity has been monitored in stable transgenic zebrafish assays [33], [34], [35]. Finally, we show that a fraction of the HCNR for which we did not detect enhancer activity in F0 assays behave as enhancer blockers in vivo.

## Materials and Methods

### Ethic statement

Zebrafish transgenic fishes have been maintained at the CABD Animal Facility. Our Animal Facility in accordance with nacional and European regulations is registered as animal research center with the number SE/4/U. Veterinary welfare supervision and daily water check-ups are conducted (dissolved oxygen, conductivity, pH, ammonia, nitrites, nitrates, alkalinity and hardness –Kh and Gh-, among other parameters) to ensure the animals good health status. Temperature, humidity and light intensity control in the room are strictly monitorized to guarantee animal welfare. Zebrafish embryos have been sacrificed after being anesthetized with 0.016% tricaine when necessary. The experimental zebrafish procedures have been performed following the protocols approved by the Ethical Committee for Animal Research from Consejo Superior de Investigaciones (CSIC) according to the European Union regulations.

### Animal care

Zebrafish (*Danio rerio*) were maintained and obtained from our breeding colony under standard conditions according to previously stated procedures (http://zfin.org). Embryos for Tol2 transgenesis were obtained from crosses of wild-type AB/Tuebingen (AB/TU) zebrafish. Potential transgenic founders were out-crossed to a TAP strain. Fertilized eggs were kept at 28°C in E3 medium with 0.003% 1-phenyl-2-thiourea to prevent pigmentation and were staged according to Kimmel et al. [36].

### ZED-HCNR Collection

Human HCNR fragments where amplified using HiFi Taq polymerase (Roche, Manheim, Germany) using standard PCR procedures. Products where cloned into pCR8/GW/TOPO vector (Invitrogen, Pasadena, USA). HCNR-containing clones where recombined into the Zebrafish Enhancer Detection (ZED) shuttle transgenesis vector previously described [32]. Briefly, ZED-Vector contains two modules flanked by the Medaka (*Oryza latipes*) Tol2 transposase target sites, that enables an efficient transgenesis [37]. The first module contains the minimal GATA promoter driving the expression of the enhanced green fluorescent protein (EGFP). All HCNRs were cloned upstream of this module using the Gateway system (Invitrogen, Pasadena, USA). Two strong insulators, which reduce the potential influence of the regulatory elements that may be present in the vicinity of the integration sites, flank this reporter cassette. The second module contains the cardiac actin promoter driving the expression of the red fluorescent protein (RFP), which serves as a positive control for transgenesis in F0 and F1 embryos [32]. The tested HCNRs are listed in Table S1.

### Selection of enhancer-containing HCNRs candidates

A minimum of 300 embryos where injected with 3–5 nl of a solution containing 25 nM of each construct and 25 nM of Tol2 mRNA. Embryos where then incubated at 28°C as previously described. EGFP expression was evaluated 24, 48 and 72 hours post-fertilization (hpf). Whenever EGFP was observed, the HCNR tested was considered as a potential candidate and embryos were selected and raised to sexual maturity to be analyzed in F_1_. The efficiency of the integration of the ZED-HCNR construct in the injected embryos was determined by the expression of RFP in the somites and the heart. We only evaluated the enhancer potential of the HCNRs when RFP was broadly observed in the somites and the heart of the injected embryos, as an indication of efficient ZED-HCNR integration. For high-resolution pictures a F-View black/white digital camera coupled to a WD70 Nikon camera was used. Adobe Photoshop was used to adjust bright and contrast.

### Enhancer-blocking assays

To evaluate *in vivo* a potential insulator activity of HCNRs, we used a Tol2 vector previously described [32]. This construct contains a strong midbrain enhancer, a Gateway entry site and the cardiac actin promoter controlling the expression of EGFP. Each candidate HCNR was recombined between the midbrain enhancer and the cardiac actin promoter (INS-HCNR). As a reference, the empty backbone was used (INS-zero). One cell-stage embryos where injected with 3–5 nl of a solution containing 25 nM of each construct plus 25 nM of Tol2 mRNA. Embryos where then incubated at 28°C and EGFP expression was evaluated 24 hpf. The midbrain/somites EGFP intensity ratio was quantified using ImageJ freeware and was directly proportional to the enhancer-blocking capacity. As a positive control, the chicken beta-globin insulator 5HS4 was used. Each experiment was repeated independently and double-blinded to the operators.

## Results

### Enhancer activity of human HCNRs in zebrafish embryos

**Figure 1:**
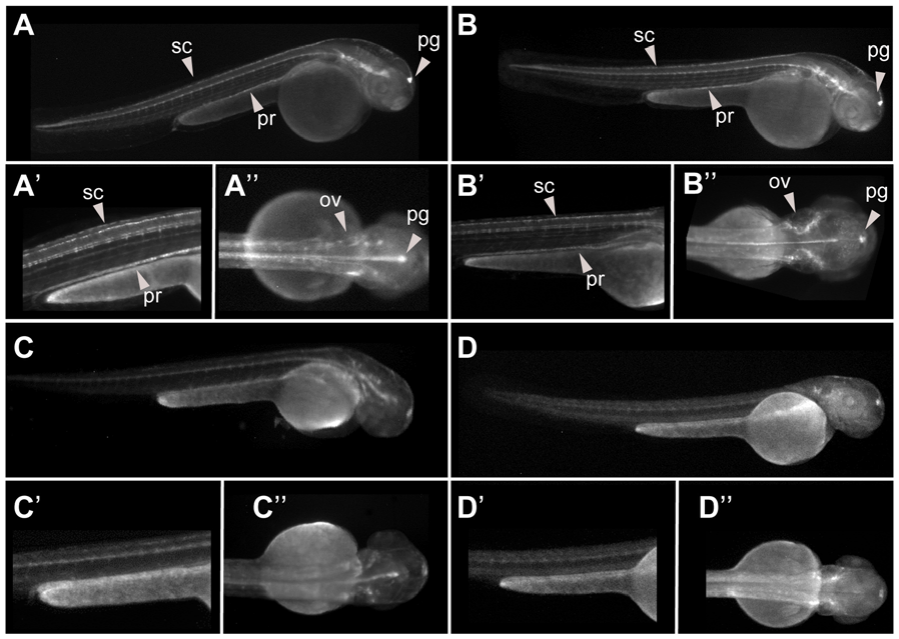
Reproducible enhancer. EGFP expression patterns exhibited from four different founders f(A–D) of the HCNR C32 at 48 hpf. EGFP expression can be seen in otic vesicle (ov), spinal cord (sc) and pronephros (pr). Fluorescence in the pineal gland (pg) in these and other embryos shown below correspond to non specific expression observed in most transgenic generated with the ZED vector.

**Figure 2:**
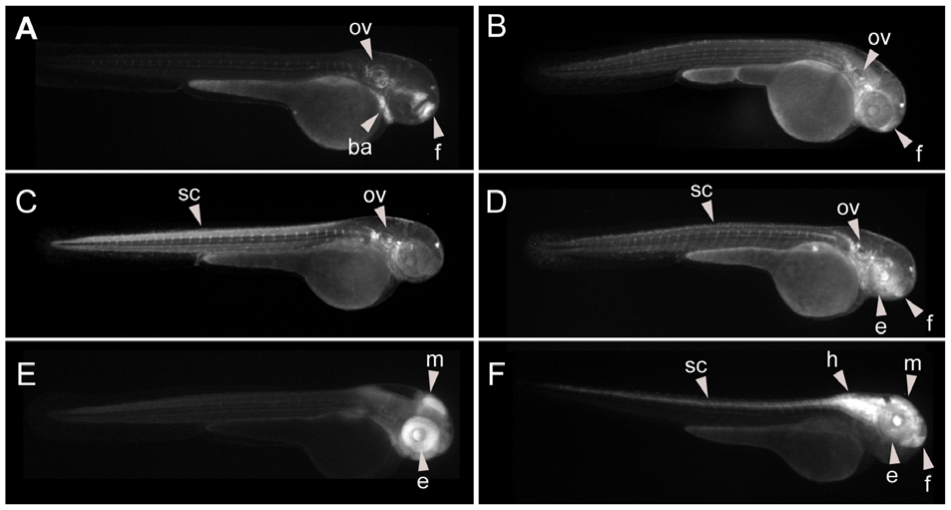
CNR with genomic boosting behaviour. EGFP expression patterns exhibited from six different founders (A–F) from HCNR C60 at 48 hpf. EGFP expression can detected in different territories depending on the founder, suggesting that a transcription pattern largely depending on the genomic context. Abbreviations are: branchial arches (ba), otic vesicle (ov), eye (e), forebrain (f), midbrain (m), hindbrain (h) and spinal cord (sc).

A total of 113 HCNRs from the human chromosome 16 were PCR-amplified, transferred to the ZED vector to generate the corresponding ZED-HCNR constructs, and injected in zebrafish embryos (Table S1). Among them, 39 (34%) exhibited mosaic EGFP expression at 24, 48 and/or 72 hpf in F0 and where therefore selected for their analysis in F1 stable transgenic lines. The remaining constructs did not show visible EGFP activity although clear and homogenous RFP expression in the somites and heart was observed, indicating an efficient integration of the cassette. In order to determine the ratio of false negatives, 10 random HCNRs with no apparent F0 EGFP activity were also raised to sexual maturity and screened for enhancer activity in stable transgenics. Finally, to determine the likelihood of enhancer trapping of our reporter cassette, the empty ZED vector without any cloned HCNR upstream the minimal promoter (ZED-zero) was also injected and the embryos grown to sexual maturity. Upon raising and out-crossing the adult fishes, 35 HCNRs were suitable for analysis. For the remaining ones we only obtained a single founder that precludes us to unambiguously determine the real enhancer activity of the HCNR under evaluation. A first analysis highlighted that approximately 63% of the F0 EGFP^+^ HCNRs (22 out of 35) do showed enhancer activity in stable lines. The expression patterns promoted at different tissues in the different founders of each HCNR are summarized in Table 1. Among these 22 regions, 9 HCNR showed reproducible expression patterns among founders (Fig. 1 and Fig. S1). These HCNRs were considered to contain robust enhancers. The remaining 13 HCNRs contained enhancers with more variable activity observed between their corresponding founders (Table 1 and Fig. S2). A similar proportion between enhancers with robust and variable activities has been shown before when assaying HCNRs from other genomic regions [33], [34], [35]. From these 13 HCNRs, we found one extreme case in which the different founders showed strong but largely non-overlapping expression patterns (Fig. 2). This phenomenon has been traditionally named enhancer trapping. However, according to the vector design, the two strong insulators flanking the expression cassette should reduce unspecific EGFP expression caused by the genomic context in which the integration occurs. Indeed, among six independent founders containing the empty ZED vector only two showed some weak position effect (Fig. S3), which confirmed that our reported construct prevents strong position effects. Therefore, the HCNR showing multiple founders with strong but different expression patterns seems likely overcoming the influence of the insulators of the reporter module and boosting the enhancer activity of the genomic landscapes around each particular transgene insertion point. Interestingly, we have also detected this type of booster activity in other regulatory regions found within other unrelated HCNR enhancer screens (unpublished results).

**Table 1:** Enhancer activity displayed by the different HCNRs assayed.

Construct	GFP in F0	Founders (n)	Notochord	Neural tube	Forebrain	Midbrain	Hindbrain	Otic vesicle	Pronefros	Eye	Notes
C25	+	3	2	1	0	1	1	0	0	0	
C29	+	5	0	3	0	0	2	3	4	0	
C30	+	3	0	1	3	3	3	2	0	0	
C32	+	3	0	3	0	0	0	3	3	0	
C33	+	3	0	1	2	0	1	2	0	0	1 Somites.
C36A	+	3	1	0	0	0	1	0	0	0	At 24 hpf.
C40	+	4	0	1	2	0	1	2	0	1	
C41	+	3	0	3	3	3	3	3	0	0	
C43	+	3	0	0	0	0	0	0	0	0	
C44	+	5	1	2	2	2	2	0	0	0	
C50A	+	3	1	1	2	2	1	1	0	0	
C60	+	8	0	2	4	2	2	5	0	1	
C61	+	5	0	0	0	0	0	0	0	1	1 Somites at 24 hpf.
C76	+	4	0	1	1	1	1	0	0	1	1 Pectoral fin.
C81	+	3	0	2	3	3	3	2	0	3	1 somites at 24 hpf.
C86	+	5	0	1	0	1	1	2	0	3	
C91	+	2	0	0	0	0	0	0	0	0	
C96	+	4	0	1	1	0	0	1	0	1	
C97	+	7	0	0	4	0	0	2	0	1	
C106	+	3	0	0	0	0	0	1	0	1	
C107	+	3	0	0	0	0	0	0	0	0	1 Hind/Midbrain boundary.
C114	+	2	0	2	2	1	1	1	0	0	
C118	+	3	0	1	0	1	1	1	0	0	
C121	+	4	0	0	0	1	1	2	0	1	
C122	+	3	0	0	0	1	1	2	0	1	
C130	+	2	0	0	0	0	2	1	0	0	Expression at 24 hpf.
C134A	+	7	0	0	0	0	2	0	0	0	
C135	+	3	1	2	0	0	0	3	0	0	1 Hutching gland.
C137	+	5	0	4	0	0	2	0	0	1	
C139	+	3	0	0	0	0	3	0	0	0	
C140	+	8	0	2	1	0	0	0	0	0	
C141	+	3	0	0	0	0	3	0	0	3	
C145	+	2	0	2	2	2	2	0	0	0	
C150	+	2	0	0	0	0	0	0	0	0	
C153	+	4	0	2	2	0	2	3	0	0	
C26	−	3	0	0	0	0	0	0	0	0	
C35	−	8	0	0	2	0	1	1	1	1	
C52	−	2	0	0	0	0	0	0	0	0	
C59	−	4	0	1	2	0	2	2	0	1	
C65	−	2	0	1	0	1	1	0	0	0	
C78	−	2	0	0	0	0	0	0	0	0	at 24 h, general expression.
C82	−	2	2	0	2	0	1	2	0	0	
C90	−	2	0	0	0	0	0	0	0	0	
C99	−	3	0	0	0	1	0	0	0	0	
C111C	−	3	0	0	1	0	0	0	0	1	1 gut.

Finally, among the 10 HCNRs that were EGFP^−^ in F0 assays and were surveyed for enhancer activity in F1 stable lines, 8 of them exhibited only the RFP expression corresponding to the positive control contained in the vector. However, the remaining two (C82 and C59; Table 1, Fig. S1 and Fig. S2) did contain enhancer activity.

### Transient versus stable transgenic assays

**Figure 3:**
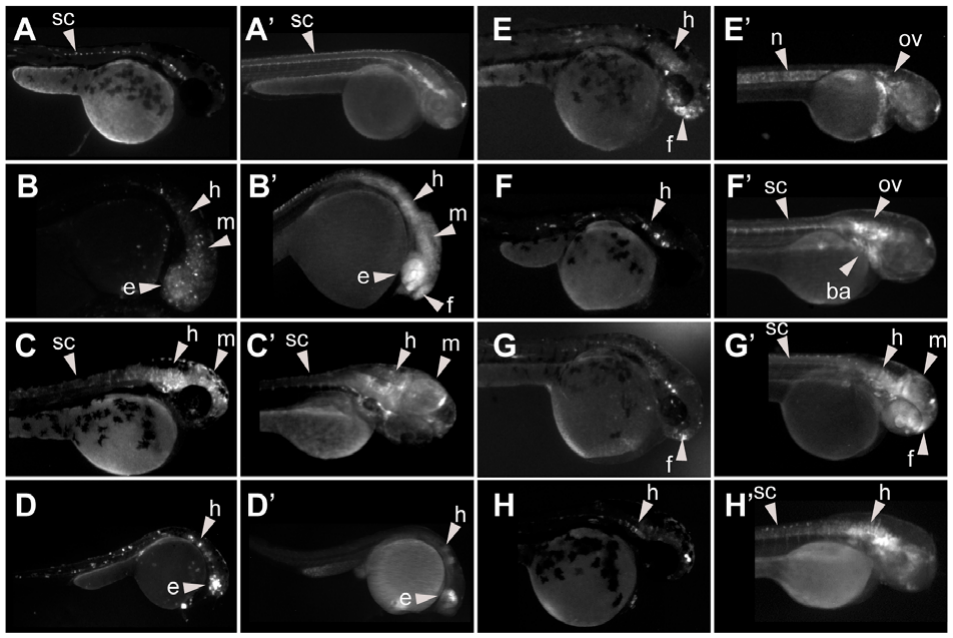
Reproducible enhancers may be distinguished in F0. Side by side comparison of the expression patterns expected from F0 (left panels) and the corresponding F_1_ (right panels). Strongly (A–D), but not weakly (E–H) reproducible enhancers showed a high similarity in transient (A–H) and stable (A′–H′) transgenic assays. Abbreviations are: notochord (n), branchial arches (ba), otic vesicle (ov), eye (e), forebrain (f), midbrain (m), hindbrain (h) and spinal cord (sc).

Many groups use the compilation of the results from several mosaic transient transgenic embryos to extract the regulatory potential of a candidate regulatory element, assuming that this compilation would recapitulate the expression that should be observed in stable transgenic lines [7], [24], [29], [30], [31]. This type of experimental approximation is particularly interesting given the fact that most of the effort required for the generation of transgenic zebrafish animals resides in raising and out crossing the injected fishes. In our screening, we have documented the enhancer activity of all HCNRs in F0 injected embryos and generated stable lines for all potential enhancer regions positive in these transient assays. This has allowed us to compare the enhancer behavior of HCNRs in F0 and F1 trasngenic embryos. Our results indicate that for those HCNRs with reproducible enhancer activity in F1 stable lines, F0 data would be a good predictor for expression patterns in F1, being always the information obtained from stable lines more compete (Fig. 3A–D). In contrast, transient F0 are poor predictors of patterns driven by less-specific enhancers (Fig. 3E–H). This, along with the fact that F0 negative regions, in some cases, do show enhancer activity in F1 stable lines, indicate that conclusions drawn from enhancer analysis in F0 transient assays may be incomplete and in cases misleading.

### Comparison of HCNR enhancer activity in mice and zebrafish embryos

**Figure 4:**
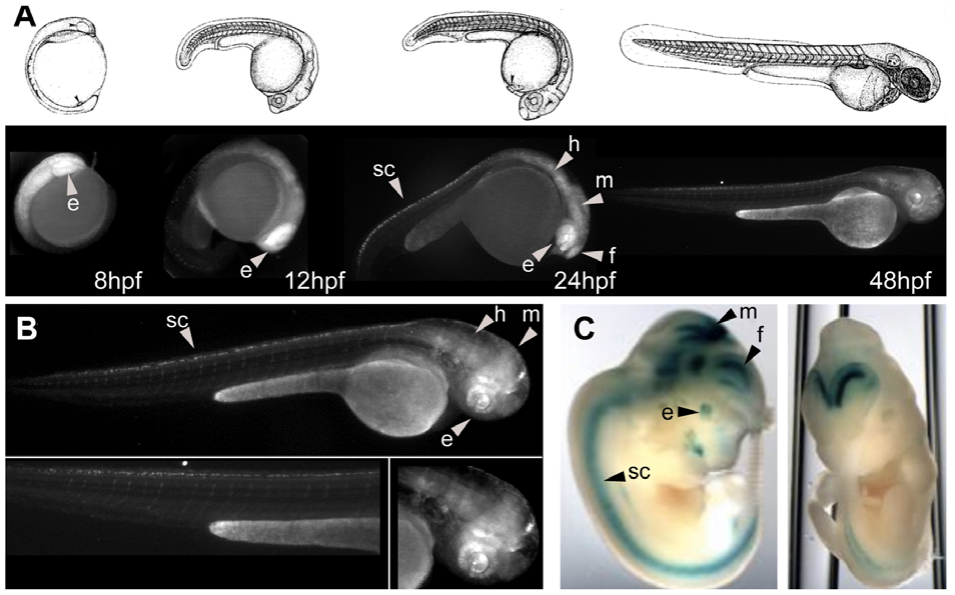
Enhancer assays in zebrafish allow a detailed spatiotemporal characterization of the expression patterns. A) Illustration of the first 48 hpf development of the zebrafish (upper panel). In the lower panel, EGFP expression of HCNR C81 during the first 48 hpf. B) Detailed EGFP expression of the HCNR C81 at 48 hpf. The same CNR was as assayed (Vista browser element37, http://pipeline.lbl.gov/cgi-bin/gateway2). Abbreviations are: eye (e), forebrain (f), midbrain (m), hindbrain (h) and spinal cord (sc).

We have also compare our results with those produced in mice and available at public databases (http://enhancer.lbl.gov/, [5]. We found 6 human sequences with tissue-specific enhancer activity in mice that partially overlapped our initial HCNR collection. Three of them were also detected as enhancers driving consistent tissue-specific patterns in our zebrafish assays (C81, C139, C141, table 2). The expression patterns observed in zebrafish embryos were similar to that observed in mouse embryos (Fig. 4, Fig. S4 and Fig. S5), suggesting that the transcription factors required to activate these enhancers are similarly expressed in both mice and zebrafish.

**Table 2:** Comparison of the enhancer activity among the HCNRs assayed in the present study *versus* those available from VISTA.

VISTA	Chr	Start	End	size	Activity	Present Study	Chr	Start	End	Size	Activity	Overlapping (bp)
element73	16	50134226	50135602	1376	+	C48	16	50134268	50135590	1322	−	1322
element65	16	50515789	50517189	1400	+	C55	16	50516006	50516943	937	n.a.	937
element37	16	53208099	53209383	1284	+	C81	16	53208063	53209682	1619	+	1284
element27	16	53636738	53637930	1192	+	C90	16	53636501	53637749	1248	n.a.	1011
element23	16	53981917	53983239	1322	+	C93	16	53981929	53983259	1330	−	1310
element16	16	71538401	71540046	1645	+	C109	16	71539086	71539495	409	−	409
element4	16	78930094	78931256	1162	+	C139	16	78930617	78931252	635	+	635
element1	16	84987588	84988227	639	+	C141	16	84987535	84988024	489	+	436

The other three enhancers active in mice cases were found negative in our F0 zebrafish assays and therefore not selected for F1 analysis (C48, C93 and C103). It is possible that, since the exact sequence included in the constructs for the two experiments was not the same, sequence differences might account for the different experimental outcome. Alternatively, these sequences might have behaved as negative in transient but have shown activity if established as stable lines. Finally, all HCNR detected as enhancer in zebrafish had been shown to be enhancers in mice as well.

### HCNRs negative in enhancer assays may harbor insulators

**Figure 5:**
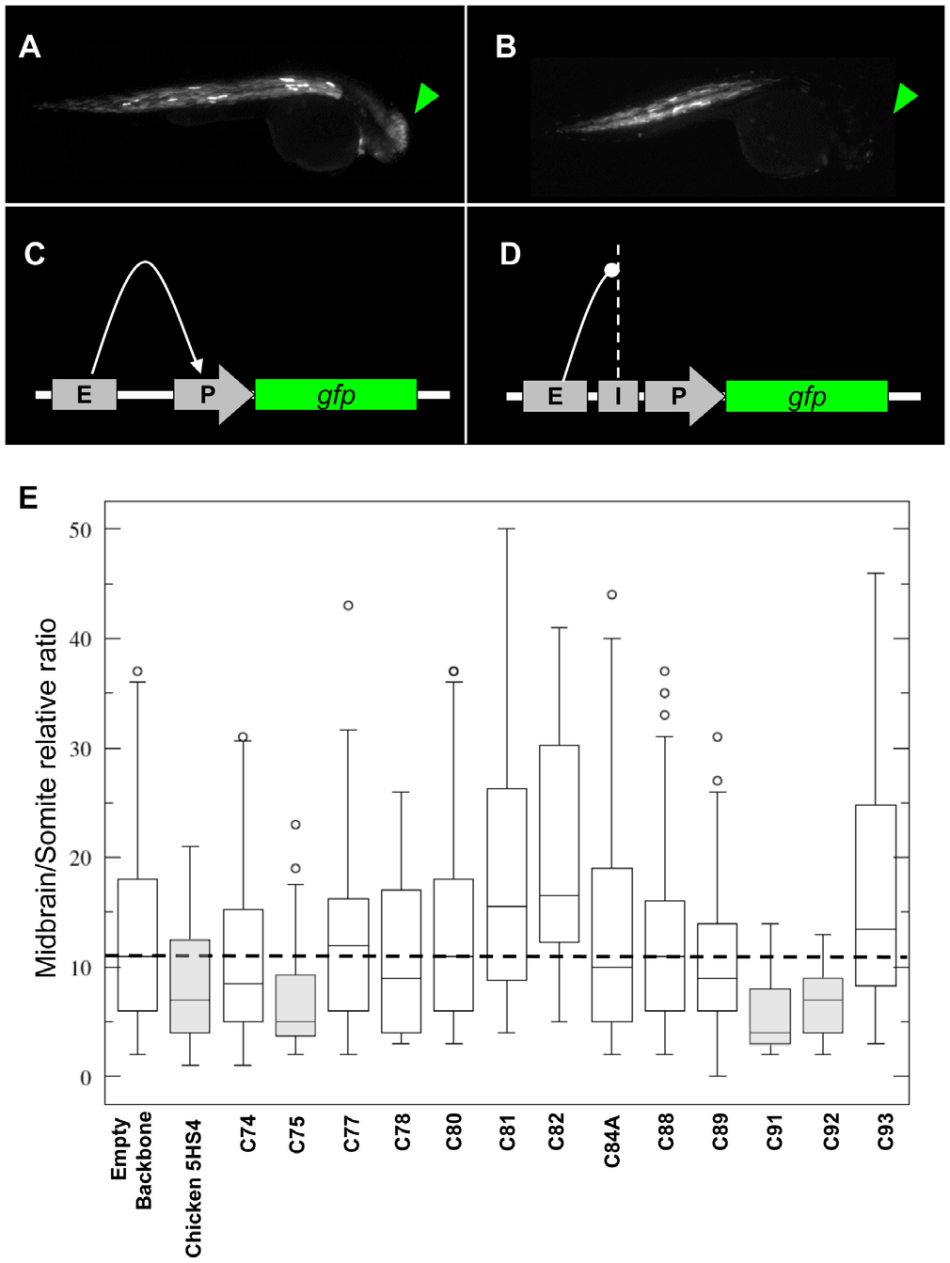
*In vivo* enhancer-blocking assay of HCNRs mapping the human *IRXB* locus. 30 hpf zebrafish injected with the insulator-vector assay lacking any HCNR (panel A) and C91 (panel B). With no insulator activity, Z48 enhancer interacts with the cardiac actin promoter promoting EGFP expression to the midbrain (C). Whenever an insulator is placed between the enhancer and the promoter, midbrain expression is reduced when compared to the somites expression, which remain unaffected. Adapted from Bessa et al, 2009. E) Wisker-plot representation of the midbrain/somite ratios from different regions tested.

Among the different types of cis-regulatory elements, insulators play key roles in controlling gene expression and organizing the chromatin [38]. Since many HCNRs did not showed enhancer activity, we determined if a fraction of them could be associated with insulators activity. For that we used a recently described vector [32] that has been used in zebrafish to functionally evaluate insulator activity *in vivo* [32], [39], [40]. We concentrated on 13 HCNRs lacking enhancer activity in our initial F0 enhancer assays and located all along 2 Mb covering the *Iroquois B* (*IRXB*) genomic cluster. Interestingly, three of these HCNRs showed a significant enhancer-blocking activity, ranging between 40–60% blockage (C75 and C91, respectively. p<10^−3^, student t-test) (Fig. 5, Table S2). These data suggest that HCNRs, in addition to harboring enhancer elements, also contain insulators that regulate enhancer-promoter interactions.

## Discussion

In this report we present a chromosome-wide analysis of the HCNRs present on human chromosome 16. Among the 113 HCNRs assayed, 35% showed enhancer activity in transient F0 transgenic embryos. Nevertheless, only 60% of them are associated with detectable enhancer activity in stable (F1) zebrafish transgenic lines. Only those enhancers showing highly reproducible expression in F0 transient assays correspond to those that are also highly reproducible in stable lines. Therefore, F0 assays are only informative for strong enhancers. Indeed, here we show that 40% of HNCRs scored positive in F0 transient assays may not be real enhancers. Most of these regions showed a low number of EGFP positive cells in the F0 assays, which may indeed reflect positional effects and not true enhancer activity. In addition, by examining in stable transgenic lines the activity of a fraction of the regions scored negative in F0 assays, we showed that 20% of them display enhancer activity. Therefore, at least with our ZED vector and using human sequences, F0 transient transgenic zebrafish assays might be unreliable as predictors of enhancer activity, as we detect 40% of false positives and 20% of false negatives. A similar analysis to that performed here would be recommended for other vectors commonly used for evaluating enhancers in zebrafish though F0 transient assays to determine their specificity and sensitivity.

We have generated multiple different founders for the 24 enhancers we have identified. This allows us to categorize the enhancer activity of the HCNRs in two major groups: highly reproducible enhancers and less specific ones, as previously also described [33], [34], [35]. Within the last group, we find one HCNR with apparently strong booster activity: Different founders for this region show strong but unrelated expression patterns. This is something that we have also observed for other enhancers previously identified (unpublished results). Indeed, this type of very interesting regions, although barely characterized, has been previously described for the mice TAL1 gene [41]. In this work, a mammalian interspersed repetitive element (MIR) was shown to boost the activity of a close enhancer. Acting together, both enhancer and booster drive expression of TAL1 to different hematopoietic tissues in transgenic mice. The HCNR with booster activity we now identify is located within a gene desert between human *SALL1* and *TOX3* genes. Eight independent founders provide evidence that this region may be playing a positive role over transcription, however its physiological role and its target gene are still unknown.

Cis-regulatory elements include enhancers, silencers, insulators and likely other unidentified type of sequences [42]. All of these types of elements could be in principle highly conserved at the sequence level in the vertebrate lineage [43]. However, to our knowledge, HCNRs have been only functionally assayed for enhancer activity. We show that 20% (3 of 13) of the HCNRs examined, that do not show any enhancer activity in F0 transient assays, seem to behave as insulators. This strongly indicates that functions other than enhancer activity is associated also with highly conserved sequences. We have examined the region comprising the *Iroquois B* (*IRXB*) genomic cluster, an evolutionary conserved cluster that spans ≈1.3 Mb of the chromosome that contains three developmental genes (*IRX3*, *5* and *6*) with multiple function during development [44]. To be able to exert these multiple functions, these genes have complex expression patterns [45], [46], [47] controlled by multiple cis-regulatory elements spread all over the cluster, many of them located within HCNRs [6]. These cis-regulatory sequences precisely interact with their respective target promoters depending on the three-dimensional looping of the cluster's chromatin [22]. The *IRXB* region contains a significant enrichment of HCNRs when compared to the rest of the chromosome (2% of the chromosome's size harboring 20% of the total HCNRs), which correlates with the highly complex regulation of the genes within it [6], [22]. The high proportion of sequences with insulator activity in this region may be thus associated with the complex regulation of the *IRXB* genes. It remains to be determined if a similar fraction of insulator also exists in HCNRs from other chromosomal regions.

Most insulators found in vertebrates are associated with the DNA binding factor CTCF [48]. When HCNRs with insulator function where subjected to *in silico* motif discovery for CTCF, these sequences exhibited weak scores according to the position weigh matrix tested. Moreover, the examination of the available data on the distribution of CTCF in different human cell lines generated by the ENCODE project [13] and available at the UCSC browser [49], also indicated that these HCNRs are not bound by CTCF in those cell lines. Therefore, it is likely that additional insulator-associated proteins may be responsible for the enhancer-blocking activity displayed by these sequences.

In summary, our large enhancer screen allows us to show the different types of enhancer activities within HCNRs, ranging from very specific and reproducible enhancers to boosters with little tissue-specificity. In addition, for the first time, we have uncovered the presence of insulator activity within these conserved sequences. Many other functions such as some required for chromatin topology or repressor activities could be also associated to these HCNRs. Indeed, many HCNRs did not behave either as enhancers or as insulators in our functional assays. However, the identification of such activities remains a future challenge.

## Supporting Information

Figure S1 **Expression patterns associated to HCNRs containing robust enhancers.** Each box contains a series of pictures showing the expression pattern obtained from different founders from a single HCNR. Pictures were taken using a black/white camera with a GFP filter.
(TIF)

Figure S2 **Expression patterns associated to HCNRs with variable enhancer activity.** Each box contains a series of pictures showing the expression pattern obtained from different founders from a single HCNR. Pictures were taken using a black/white camera with a GFP filter.
(TIF)

Figure S3 **Controls suggest a low enhancer trapping capacity of the empty ZED vector.** Diagram showing the structure of the ZED vector (A). Transgenic zebrafish evaluated at 48 hpf from six independent founders obtained from the ZED-zero construct. Pictures evidenced both spurious or no EGFP expression (B–F), despite strong RFP expression in the somites (G). Abbreviations: Tol2: Tol2 transposase target site; C. Actin: cardiac actin promoter; rfp; red fruorescent protein gene; ins: insulator; gfp: green fluorescent protein gene; Min. Prom: minimal promoter; entry site: gateway entry site, which was eliminated to generate the ZED-zero construct.
(TIF)

Figure S4 **Comparison of the enhancer activity determined for C139** ***versus*** **the data available from VISTA Element-4.** Three different founders from zebrafish (A) and mice (B), obtained upon the evaluation of the enhancer activity of the human sequence assigned as C139 (A), or Element-4 (B). In panel C we represent the alignment of both sequences.
(TIF)

Figure S5 **Comparison of the enhancer activity determined for C141** ***versus*** **the data available from VISTA Element-1.** Three different founders from zebrafish (A) and mice (B), obtained upon the evaluation of the enhancer activity of the human sequence assigned as C141 (A), or Element-1 (B). In panel C we represent the alignment of both sequences.
(TIF)

Table S1 **Details of the highly conserved non-coding regions assayed.**
(DOC)

Table S2 **In vivo enhancer-blocking assays.**
(DOC)
