# Supplementary material for: Dissecting the Transcriptional Regulatory Properties of Human Chromosome 16 Highly Conserved Non-Coding Regions
Source: PLoS One. 2011 Sep 13;6(9):e24824. doi: 10.1371/journal.pone.0024824 (PMC3172297; doi:10.1371/journal.pone.0024824)
Supplement: Table S2 — In vivo enhancer-blocking assays. (DOC) [file pone.0024824.s008.tif]

# Supporting Table II. *In vivo* enhancer-blocking assays

|  | n | Mean [min-max] | SD | p-value |
| --- | --- | --- | --- | --- |
| Empty backbone | 55 | 12.5 [2-37] | 7.82 | - |
| Chicken_5HS4 | 25 | 8.16 [1-21] | 5.26 | 0.013 |
| C74 | 42 | 10.8 [1-31] | 7.77 | 0.29 |
| C75 | 46 | 7.02 [2-23] | 4.66 | <0.001 |
| C77 | 46 | 12.7 [2-43] | 8.46 | 0.92 |
| C78 | 33 | 11.0 [3-26] | 7.24 | 0.36 |
| C80 | 70 | 13.5 [3-37] | 9.56 | 0.54 |
| C82 | 50 | 19.5 [4-50] | 12.8 | 0.001 |
| C83 | 32 | 20.3 [5-41] | 10.3 | <0.001 |
| C84A | 67 | 13.1 [2-44] | 9.65 | 0.73 |
| C88 | 94 | 12.3 [2-37] | 7.65 | 0.85 |
| C89 | 65 | 10.6 [1-31] | 6.53 | 0.16 |
| C91 | 31 | 5.9 [2-14] | 3.74 | <0.001 |
| C92 | 31 | 6.42 [2-13] | 2.90 | <0.001 |
| C93 | 36 | 17.6 [3-46] | 12.0 | 0.017 |
